# Effects of developmental exposure to pesticides in wax and pollen on honey bee (*Apis mellifera*) queen reproductive phenotypes

**DOI:** 10.1038/s41598-020-80446-3

**Published:** 2021-01-13

**Authors:** Joseph P. Milone, David R. Tarpy

**Affiliations:** 1 Department of Entomology and Plant Pathology, North Carolina State University, Raleigh, NC 27695 USA; 2 Biology Graduate Program, Ecology and Evolution, North Carolina State University, Raleigh, NC 27695 USA

**Keywords:** Entomology, Physiology

## Abstract

Stressful conditions during development can have sub-lethal consequences on organisms aside from mortality. Using previously reported in-hive residues from commercial colonies, we examined how multi-pesticide exposure can influence honey bee (*Apis mellifera*) queen health. We reared queens in beeswax cups with or without a pesticide treatment within colonies exposed to treated or untreated pollen supplement. Following rearing, queens were open-mated and then placed into standard hive equipment in an “artificial swarm” to measure subsequent colony growth. Our treated wax had a pesticide Hazard Quotient comparable to the average in beeswax from commercial colonies, and it had no measurable effects on queen phenotype. Conversely, colonies exposed to pesticide-treated pollen had a reduced capacity for viable queen production, and among surviving queens from these colonies we observed lower sperm viability. We found no difference in queen mating number across treatments. Moreover, we measured lower brood viability in colonies later established by queens reared in treated-pollen colonies. Interestingly, royal jelly from colonies exposed to treated pollen contained negligible pesticide residues, suggesting the indirect social consequences of colony-level pesticide exposure on queen quality. These findings highlight how conditions during developmental can impact queens long into adulthood, and that colony-level pesticide exposure may do so indirectly.

## Introduction

The combined contributions of the genome and environment on human health highlights the importance of characterizing exposures and their impacts in other organisms^1^. The exposome encompasses all of the chemical, physical, and biological agents experienced over a lifetime including during sensitive developmental stages^2^. Recently, an increased emphasis has been placed on the fetal origins of disease as it has been found that developmental conditions, including chemical exposures and undernutrition in utero*,* can dictate latent health outcomes during adulthood^3,4^. For example, exposure to endocrine disrupters during sensitive developmental windows has been linked to metabolic disturbances later in life, such as diabetes and obesity^3^. Additionally, chemical exposures of mature adults can result in perturbations of both male and female reproductive physiology as well as transgenerational epigenetic impacts^3^. Invertebrates often share similar responses to chemical exposures and as a result have been used widely to model their effects on vertebrate development and reproduction^5^.

Honey bees (*Apis mellifera*) are not only an economically important crop pollinator^6^ but are also an alternative model system for examining the underlying concepts forming the human exposome. Whereas the vast majority of insect toxicology has emphasized understanding chemical lethality with the aim of pest management, pollinator toxicology is tasked with characterizing risk for the protection of beneficial insects. This has broadened honey bee pesticide research to incorporate components analogous with human toxicology, such as sublethal and chronic exposures, reproductive and developmental impacts, and multi-stressor interactions^7,8^. While human research is typically observational and correlative, honey bees can be subjected to manipulated exposure environments and offer a shorter-lived eusocial model to examine the impacts of chemical exposure across various life stages and social contexts.

As a eusocial “super organism”^9^, the stressors challenging an individual bee’s health can ultimately inflict a cost on overall colony fitness^10^. This is especially apparent with honey bee queens, which most directly contribute to colony fitness by monopolizing reproduction. Queens are the longest lived caste in a colony; while worker lifespans are often measured in weeks (~ 6 weeks, on average), queens can live for years (~ 1–3 years, on average) during which an individual queen can lay up to thousands of eggs in a day^11^. Moreover, queens mate with multiple males during their first week of life, and mating number can have a dramatic influence on colony phenotype because a genetically diverse worker population can help improve a colonies’ disease response and reduce inbreeding^12–14^. Queens store the sperm from their mating flights for the duration of their lives, and the ability to acquire, store, and maintain large numbers of viable sperm is key to queen longevity^15^. Queen mating health is integral to the sustainability of apiculture^16,17^, and annual surveys of commercial beekeepers in the US consistently cite queen longevity as a major factor leading to colony losses^18^. Queen losses frequently precede colony death^19^ and exposure to neonicotinoids or miticides have been found to influence queen survival, reproductive health, and immunity^20–26^.

During visits to flowers (particularly in agroecosystems), honey bee foragers can come into contact with chemicals present within their landscape and return them to their hives. As a result, pesticides can become sequestered within the honey, wax, and other internal hive matrices. Additionally, beekeepers can apply their own chemotherapies directly into a hive for the treatment of disease and parasites, which can influence a colony’s in-hive chemical environment long after their application^27,28^. Pesticide exposure can alter developmental conditions inside a hive and lead to mortal and sublethal impacts on a colony. Pesticide residue survey data, characterizing the chemical exposome within commercial honey bee colonies, has confirmed the near ubiquity of multiple pesticide exposures in honey bee colonies. Wax combs serve as the nest substrate within a colony, and it is used to rear young (brood) and store their food (honey for carbohydrates and pollen or ‘beebread’ for protein). It has been reported that beebread and beeswax in commercial beekeeping operations contain an average (± SD) of 7.2 ± 3.60 and 10.1 ± 4.90 different pesticide residues, respectively^29^. Pesticide-impregnated wax comb can facilitate pesticide transfer into other matrices (i.e., food or brood) and poses a potential risk to larvae that are exposed through contact while developing inside wax cells.

Oral exposure can occur when nurse bees consume contaminated pollen. It has been previously observed that nurse bee secretory glands can be effected during oral exposure to pesticides^30–36^, and it was recently found that pesticide-induced glandular degeneration can manifest in the quality of royal jelly (RJ) fed by nurse bees to developing queens^37^.

Conditions during sensitive early life stages can result in both immediate and downstream health implications, and developmental exposure may influence the colonies queens later establish. Queen quality is directly connected to colony fitness and thus is an important component of overall health and colony phenotype^38^. Adult exposure to pesticides has been shown to influence queen egg laying rate and brood pattern^20,39^, but the downstream implications of queen developmental conditions and their latent impacts have yet to been examined. In this study, we sought to use previously reported pesticide residue data to empirically test how multi-pesticide oral and contact exposures can influence queen health directly and the colonies they later establish.

## Methods

### Pesticide exposures

We tested pesticide treatment mixtures for wax and pollen consisting of 12 and 9 different compounds, respectively (Table 1). We chose these compounds based on residue data from a U.S. nationwide pesticide screening of commercial colonies and the mixtures consisted of frequently detected herbicides, fungicides, and insecticides at previously observed concentrations in beebread and beeswax^29^. We employed a Hazard Quotient (HQ)^40^ in order to estimate species-specific toxicity from multiple pesticides by dividing the concentration of each residue by its respective worker LD_50_ (as reported by Traynor et al.^29^). We then summed all HQs for each compound detected in a mixture in order to estimate pesticide risk. We used worker LD_50_ values when calculating HQ values in order to provide a general estimate of the toxicity of out treatments towards honey bees and to allow for better comparison with previously published residue data^29^. Additionally, LD_50_ values are not currently available for honey bee queens for most pesticides. We spiked treated wax and pollen with an added pesticide mixture, increasing their concentration, while controls did not receive any added chemical treatment other than an equal volume of solvent. We purchased all pesticides as pure technical material (≥ 95% purity) from Sigma Aldrich Inc. (St. Louis, MO) and Chem Service Inc. (West Chester, PA) and each was serially diluted in either acetone or a 1:1 acetone/hexane for pollen and wax, respectively.

**Table 1:** Target concentrations for treatments and residue detections in pollen and wax.

Pesticide	Pollen detections (ppb)			Wax detections (ppb)		
	Target	Treated pollen (actual)	Control pollen (actual)	Target	Treated wax (actual)	Control wax (actual)
Atrazine	37.3	25.0	ND	19.3	61.0	ND
Azoxystrobin	83.1	78.0	ND	22.2	56.0	12.0
Aldicarb sulfoxide	N/A	ND	ND	649	ND	ND
Bifenthrin	N/A	ND	ND	14	ND	ND
Carbaryl	364.0	368.0	ND	N/A	ND	ND
Carbendazim	N/A	TRACE	TRACE	N/A	ND	ND
Chlorothalonil	26,600.0	16,000.0	ND	53,700.0	13,000.0	ND
Chlorpyrifos	33.4	20.0	ND	68.9	113.0	ND
Coumaphos	3260.0	1870.0	ND	1755.7	1680.0	25.0
Coumaphos oxon	N/A	ND	ND	N/A	12.0	10.0
DEET	N/A	ND	ND	N/A	92.0	98.0
2,4-DMPF	N/A	9.0	14.0	43,000.0	32,900.0	4.0
Esfenvalerate	N/A	ND	ND	29.1	ND	ND
Fenpropathrin	24.6	ND	ND	74.5	ND	ND
Fluoxastrobin	N/A	ND	ND	N/A	11.0	11.0
Flutriafol	N/A	ND	ND	N/A	29.0	27.0
Fluvalinate	469.0	415.0	ND	4895.3	1100.0	53.0
Pendimethalin	143.0	105.0	ND	60.0	TRACE	ND
Thymol	N/A	89.0	ND	N/A	2200.0	2500.0

ND signifies a non-detection. Trace indicates that a compound was detected but was unable to be quantified. 2,4-DMPF and coumaphos oxon are metabolites of the pesticides amitraz and coumaphos, respectively.

#### Pollen

When making pollen supplement treatments, we pulverized commercial wildflower pollen (Glorybee Foods Inc. Eugene, Oregon) using a laboratory blender until fine and then integrated pollen powder into diet consisting of 43% crushed pollen, 43% granulated sucrose, and 14% water by mass. We used acetone as the solvent for pesticide dilutions, which comprised 1.5% of the final diet by mass. We spiked treated pollen with the pesticide mixture while control pollen received an equal amount of acetone to account for solvent effects. We thoroughly mixed all components in a 37.85L (40Qt) stainless steel bowl using an electric hand mixer (Model 62633R, Hamilton Beach, Glenn Allen, VA) and placed 40 g portions on individual wax paper sheets before storing in plastic bags at − 20 °C until use.

#### Wax

In order to make queen cups, we filtered beeswax from the previous year’s honey cappings (= newly synthesized wax) using a 5 micron filter bag to remove debris prior to pesticide impregnation. We used 1:1 acetone/hexane mixture as the solvent for pesticide dilutions in wax. We included hexane after observing its miscibility in wax and following previous studies on pesticide impregnation of beeswax^41^. We used a rotary evaporator with a 1L round bottom flask, and we set a hot water bath to 69 °C to continuously melt and mix wax flakes prior to spiking with the diluted pesticide mixture (treated) or solvent (control). The pesticide spike mixture was contained within a total volume of 8.85 ml of solvent carrier. We stored wax at − 20 °C until queen cups were produced by dipping separate tapered wooden dowels in molten wax and removing formed wax cups. We used melted untreated wax in order to affix 15 queen cell cups in alternating order between control and treated wax cups onto a common wooden grafting bar for queen rearing.

### Study site and queen rearing

**Figure 1:**
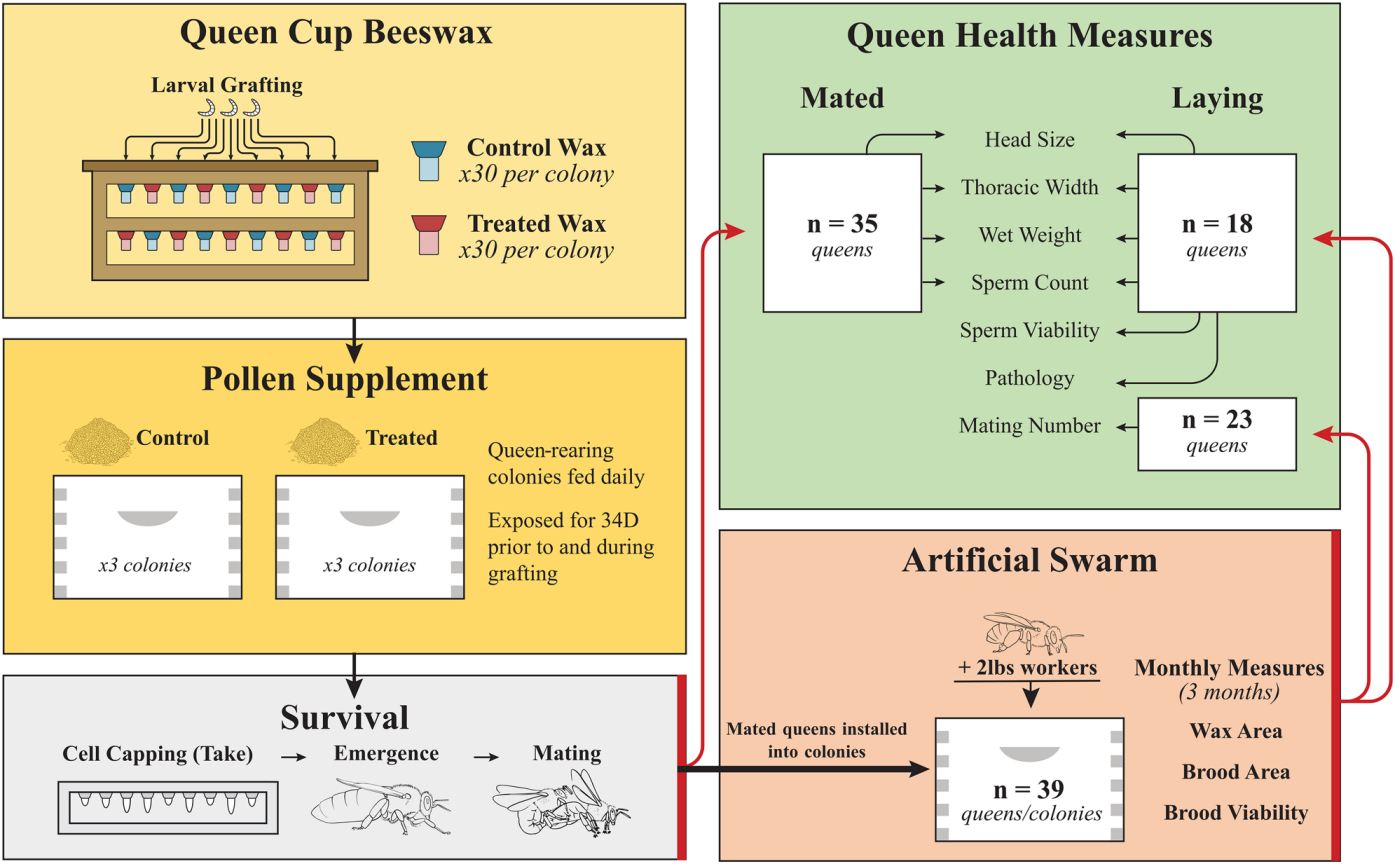
Graphical process diagram depicting experimental methods and sample sizes at different assessment stages.

We raised all queens at the Lake Wheeler Honey Bee Research Facility (Raleigh, NC) using naturally mated *A. mellifera* L. stock. We fitted pollen traps to six double-deep standard Langstroth colonies and continuously fed treated or control for 34 days prior to and during queen rearing (3 control and 3 treated colonies). Following a 24 h queenless period (“swarm box”^42^), we presented all colonies (treated and control pollen) with 60 queen wax cups (30 each of treated and control) containing 1-day old worker larvae grafted from a single source colony following the Doolittle method^42^ (see Fig. 1). After 7 days, we counted capped queen cells and placed them into an incubator set at 34.5 °C for three days. We reared queens in two consecutive grafting rounds, after which we placed them into individual mating nucleus (nuc) colonies each containing ~ 1000 worker bees for open-mating. The first and second grafting rounds were initiated 34D and 41D after starting pollen feeding. We introduced queens from the first round into mating nucleus colonies as capped queen cells, while we introduced queens from the second grafting round as caged adults following emergence within an incubator similar to Williams et al^26^.

### Queen fitness measurements

We used morphometric and sperm analysis methods adapted from Tarpy et al.^17^ to measure pesticide impacts on mated queens during two sampling events. We anesthetized queens using CO_2_ then weighed each on a digital scale to the nearest 0.1 mg. We dissected the head, thorax, and spermatheca of each queen before photographing for pixel measurement using image software (ImageJ) to the nearest 0.1 mm. Next, we used the protocol of Collins and Donoghue^43^ to determine sperm count and viability of mated queens. We crushed each spermatheca into 1 ml of Buffer D and used a pasture pipette to transfer diluted sperm into a 2 ml glass vial containing 10 µλ of both Sybr green and propidium iodide. After 5 min, we loaded 10 µλ of stained sperm into a disposable hemocytometer and used a CP-001 automated cell counter (Nexcelom biosciences, St. Lawrence, MA) to quantify live/dead counts and sperm viability.

### Mating number

We used a protocol similar to that of Withrow and Tarpy^44^ for determining the mating number of each queen through offspring microsatellite analysis. When sampling, we removed a frame of pupae from each queen’s mating nucleus, which was then immediately frozen at − 20 °C. Next, we sampled 48 individual pupae per queen and removed small piece of tissue using microscissors, which were flame sterilized between larvae, and then stored individually in 0.2 ml strip tubes. We performed DNA extractions using 150 μl 5% Chelex 100 (BioRad) in dH2O, with the addition of 5 μl 10 mg/ml Proteinase K and incubated in a thermocycler for 60 min at 55 °C, 15 min at 99 °C, 1 min at 37 °C, 15 min at 99 °C, then held at 4 °C. We used a multiplex PCR in order to amplify seven microsatellite markers (A24, A76, A88, A113, Ap43, Ap81, and B124), which have been previously used for patrilineal analysis^45,46^. We then added 1 μl of extracted DNA to a mixture of 5 μl multiplex PCR Master Mix (Kapa), 0.5 μl fluorescent-tagged primers, and 3.5 μl dH2O, then incubated in a thermocycler for 3 min at 95 °C, followed by 48 cycles of 15 s at 95 °C, 30 s at 57 °C, 45 s at 72 °C, with an elongation step of 5 min at 72 °C and held at 4 °C. Lastly, we added 1 μl of PCR products to a 9 μl solution containing Gene Scan Liz 500 sizing standard (Thermo Fisher) and Hi-Di Formamide (25 μl Liz 500 per 900 μl formamide) and then denatured for 5 min at 95 °C before being sequenced on a 3730 DNA Analyzer (Applied Biosystems) at the NCSU Genomic Sciences Laboratory (Raleigh, NC). After sequencing, we scored all peaks using Genemapper 4.0 (Applied Biosystems) then calculated the number and frequency of each subfamilies from raw microsatellite data using the program COLONY^47^, which were used to determine the observed mating number (*N*_o_) and effective mating number (*m*_e_) for each queen as described in Tarpy et al^48^.

### Colony establishment and growth measurement

We introduced a subset (N = 39) of mated queens into colonies by acclimating packages containing 2lbs of worker bees (~ 7000) from non-experimental colonies for 2 days in a dark room with a caged experimental queen. Thereafter, we installed packages into Langstroth style hive boxes each containing three frames with a 1 inch strip of wax foundation to facilitate wax comb construction. We recorded colony growth measurements over 3 months following package installation. Colonies were able to freely forage but to ensure adequate nutrition during nectar dearth periods we fed all hives the same amount of 50% sugar syrup throughout the colony growth period. After verifying all colony syrup feeders were empty, we measured colony weight using a digital game scale (Moultrie model 330) to detect changes in honey stores and worker populations. We used a 1 × 1 in. gridded wooden frame to aid in the visual measurement of nest development and wax comb construction and tracking the square inch coverage of capped brood. We also measured brood pattern solidness by counting the number of empty cells within a 3 × 3 in. square of contiguous capped brood on three randomly selected frames for each colony^49^. In late August, no more package worker bees were present in the colony and all of the workers in each colony were worker offspring from the experimental queens, at which time we sampled varroa (*Varroa destructor*) intensity within each colony using a modified “alcohol wash”^50^.

### Pathogen screening procedure

We used a Trizol RNA extraction and reverse transcription quantitative PCR (RT-qPCR) to measure commonly detected pathogens within workers from cell-building colonies before and after pollen feeding and from queens at the conclusion of the colony growth period (see Supplemental Table 5 for target primer information). We added Trizol to a Ziploc bag containing 5 g of worker bees or a vial containing a queen bee then crushed samples using either a rolling pin or a pestle and removed 100 μl of extract which was then added to 900 μl of Trizol in a separate vial. We then added 200 μl of pure chloroform and then centrifuged vials at 4 °C and 12,000G for 15 min. After removing the supernatant into a separate tube, we then added 500 μl of isoproanol centrifuged at 4 °C and 12,000G for 15 min to form an RNA pellet. We then poured off the Isopropyl alcohol and added 1 ml 75% ethanol to purify the pellet and centrifuged at 4 °C and 7500G for 5 min. Following this, we removed all ethanol and re-added 1 ml 75% ethanol prior to a final centrifugation. Lastly, we poured out all ethanol and allowed the pellet to dry before dissolving in DNASE free water in a heated dry bath at 60 °C. The RNA was then quantified and tested for purity using a NanoDrop Spectrophotometer and RNA was diluted to 200 ng/μl for all samples. cDNA was synthesized using the BioBasic all in one cDNA kit with random primers. qPCR was performed in triplicate and ran on an Applied Biosystems QuantStudio 6. Standard curves were generated using known quantities of plasmid stock of target amplicons and final results were normalized to 2 reference genes using GeNorm software (Supplemental Table 5 for primer sequences).

### Pesticide residue sampling

In order to quantify pesticide residues, we collected samples of treated and control wax and pollen and immediately placed samples on dry ice before being stored at − 80 °C. We performed a final round of grafting on each queen rearing colony in order to collect royal jelly from 3-day old uncapped queen cells 48D after initiating pollen feeding. Royal jelly was removed using a spatula while taking care not to introduce any wax into the samples. We then flash-froze samples of RJ using liquid nitrogen and stored at − 80 °C. We loaded 3 g samples into 50 ml plastic centrifuge tubes which were subsequently mailed on dry ice to the USDA-AMS National Science Laboratory in Gastonia for multi-pesticide residue screening as described in Mullin et al.^51^ using a modified QuEChERS method and analyzed by gas chromatography and liquid chromatography with mass spectrometry detection (GC/MS, GC/MS/MS, LC/MS/MS).

### Statistics

We performed all analysis using JMP Pro 14 (SAS Inc., Cary, NC). We compared daily pollen supplement consumption between colonies fed treated or untreated pollen supplement using a repeated measures mixed model with colony as a random effect, day, and the interaction between day and treatment. We made queen survival comparisons between queens in differing treatments groups using Pearson’s chi-square tests. We used paired t-tests when comparing viral loads in workers from cell builder before and after the pollen feeding period. We used standard Least Square regression for determining the impacts of wax and pollen exposure on queen physiology and sperm health. When comparing mating numbers, we used we used a t-test to compare between treatments. For colony growth comparisons, we used a repeated measures mixed model with colony as a random nested under pollen treatment with wax and pollen treatments, their interaction, and sampling month included as fixed effects. For colony brood viability, where three patches of contiguous pupal brood were assessed per colony, we used the aforementioned model with the addition of patch/rep as a random effect. We used t-tests to compare differences in mating numbers (*N*_o_ and *m*_e_) across treatments and again used t-tests when comparing differences in pathogen copy counts.

## Results

### Pesticide exposome

Pesticide residues in treated pollen and wax ranged from parts per billion to parts per million (Table 1). Treated wax used to make queen cells contained 13 different pesticides with an HQ_total_ = 2596, while control wax was found to contain low levels of 9 pesticide contaminants with an HQ_total_ = 32 (individual HQ values are shown in Supplemental Table 2). Treated pollen supplement fed to queen-rearing colonies contained 11 compounds and had a total HQ = 1653, whereas the untreated pollen supplement had low level detections of two compounds with an HQ < 1.0. Residue analysis of treated experimental matrices used for queen exposures varied relative to our target concentrations for each treatment (Table 1) and three compounds could not be detected. These could be artifacts of uneven dispersal, analytical limitations, or (in the case of wax) chemical instability during molten impregnation. Royal jelly (RJ) fed to developing queens was found to contain low levels of pesticides across treatments. However, RJ from treated wax contained the highest detected concentrations of pesticide (Supplemental Table 1). Daily pollen consumption was not different across treatment groups (F_(1,4)_ = 0.44, *P* = 0.54).

### Queen rearing and cell builder pathology

**Figure 2:**
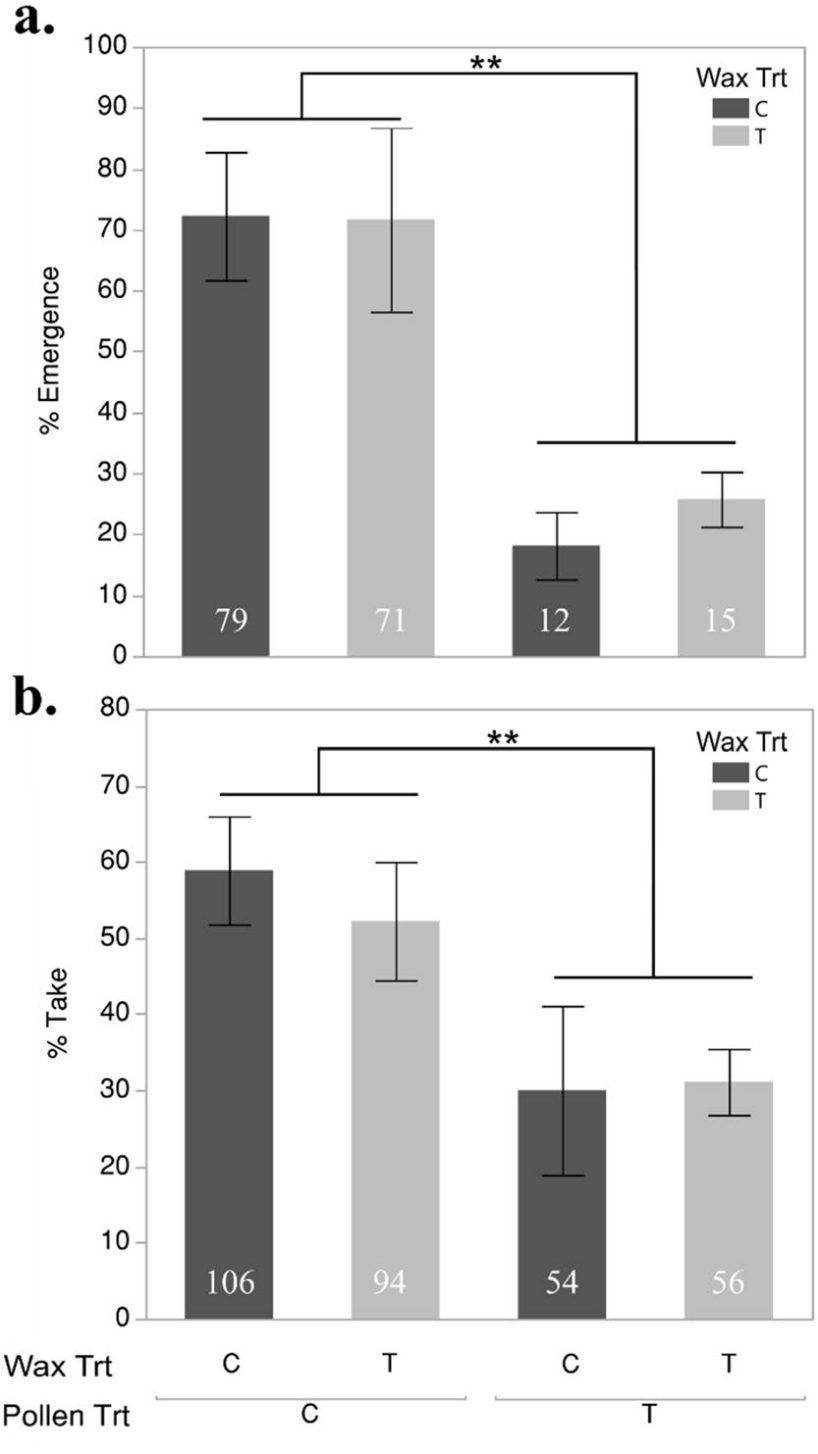
Emergence survival (**a**) and percent cell construction (% Take) (**b**) for queens reared in wax and pollen treatment (T) and control (C) environments **P < 0.0001. Sample sizes for each respective treatment are shown inside each bar. Error bars represent the standard error.

Colonies fed treated pollen supplement produced fewer capped queen cells relative to colonies fed control pollen (n = 720, Pearson χ^2^ = 45.8, *P* < 0.0001), and of the queen cells produced we observed a significant reduction in queen emergence in treated pollen supplement colonies (n = 310, Pearson χ^2^ = 73.7, *P* < 0.0001) (Fig. 2). Following the placement of virgin queens in mating nucs, there was no effect of wax or pollen treatment on the initiation of egg laying (n = 160, Pollen: Pearson χ^2^ = 2, *P* = 0.16, Wax: χ^2^ = 0.92, *P* = 0.34) or queen survival during the colony growth period (n = 39, Pollen: Pearson χ^2^ = 2.00, *P* = 0.16, Wax: χ^2^ = 0.16, *P* = 0.69). Of the three pathogens that were consistently detected in workers from cell-builder colonies, no significant differences were found when compared using a Paired t-test before and after the pollen-feeding period (Lake Sinai Virus: t_5_ = 0.52, *P* = 0.62; Trypanosomes: t_5_ = 0.90, *P* = 0.40; and *Nosema *spp.: t_5_ = -1.75, *P* = 0.14; Supplemental Table 3).

### Queen reproductive health and pathology

#### Sperm quality and viral prevalence

We analyzed a subset of queens for their reproductive quality shortly after mating and after the initiation of egg laying (n = 35), and we did the same for all surviving queens at the conclusion of the colony growth period (n = 18) (Fig. 4).Those queens sampled shortly after mating comprised the first grafting round, while queens sampled at the conclusion were from both grafting rounds. We found no significant effects of wax treatment on head width, thorax width, wet weight, or sperm count across queens reared in treated and control wax. However, differences in sperm viability were statistically significant (Whole model: F_(2,15)_ = 3.85, *P* < 0.05; Pollen treatment: F_(1,1)_ = 5.60, *P* < 0.05) when comparing laying queens reared in treated versus control pollen environments sampled at the conclusion of the experiment (Fig. 4). These same queens were found to have a large numerical difference in mean for total sperm count, and we report 4.2 × 10^6^ ± 1.8 and 2.3 × 10^6^ ± 1.5 for control and treated queens, respectively (mean ± SD). However, this difference was only marginally statistically significant (Whole Model F_(2,15)_ = 2.20, *P* = 0.14; Pollen treatment: F_(1,1)_ = 4.10, *P* = 0.06) and this was most likely the result of the reduced sample size and high variance. We detected no differences in queen wet weight, head width, or thorax width in queens from either pollen or wax treatment groups. Of the 18 queens analyzed at the end of the experiment, only half had detectable levels of pathogens (Supplemental Table 4), with the most frequent detection being Deformed Wing Virus A (50% having DWVA). No trends were observed when comparing DWVA across wax and pollen treatments.

#### Mating number

**Figure 3:**
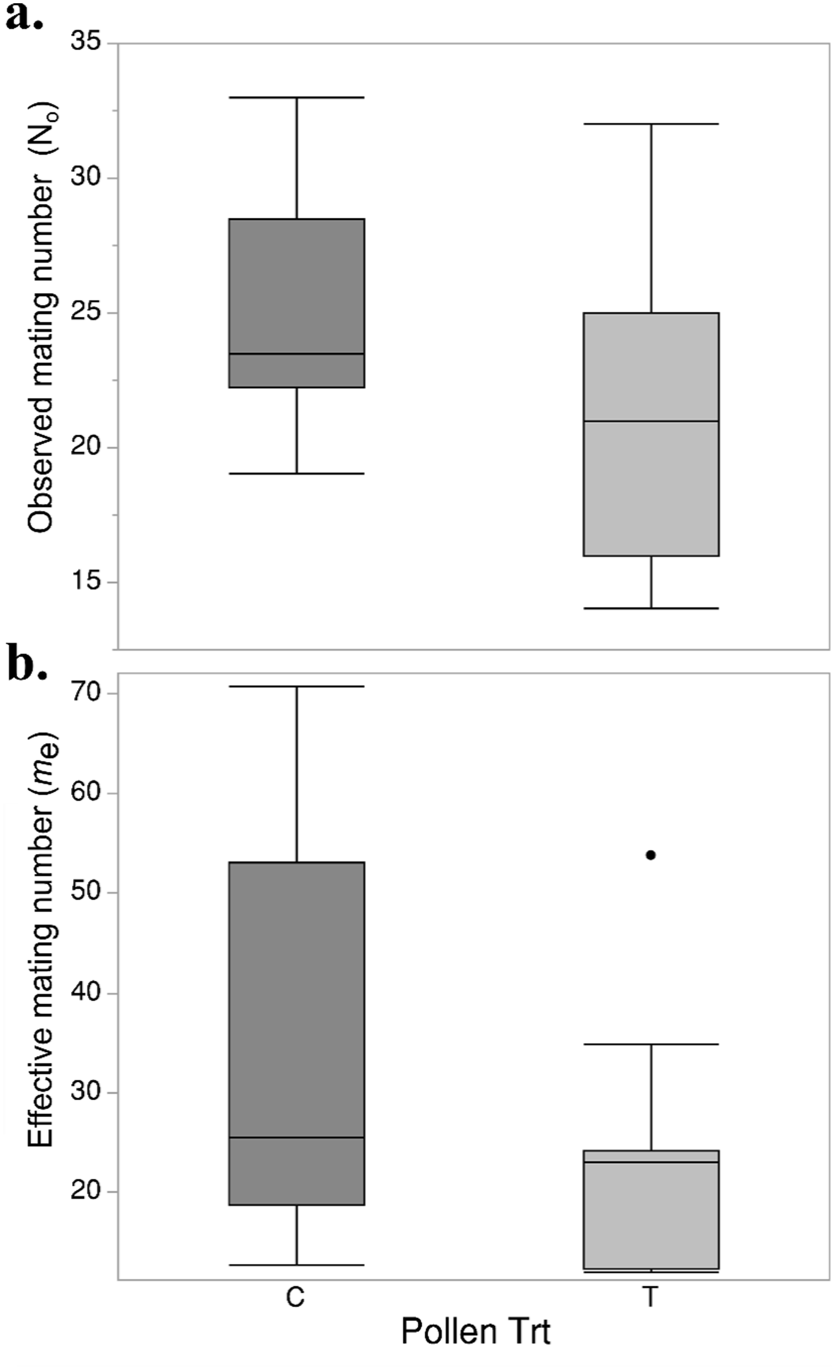
Observed mating number (**a**) and effective mating numbers (**b**) for queens reared in treated (T) (n = 11 queens) and control (C) (n = 12 queens) colonies.

In total, we analyzed the offspring from 23 queens for microsatellite paternity analysis to estimate queen mating number. We found no effect of wax treatment on the effective paternity frequency (*m*_*e*_) or observed mating number (*N*_*o*_). Queens reared in treated pollen colonies had an average *m*_*e*_ of 23.2 ± 3.69 whereas control queens had an average of 32.8 ± 5.85, but this difference was non-significant (n = 23, two-tailed t_21_ = − 1.36, *P* = 0.19). However, the pollen pesticide treatment was found to have a stronger negative trend on observed mating number (n = 23, two-tailed t_21_ = -1.897, *P* = 0.07) (see Fig. 3).

#### Colony growth

During June–August, we took monthly measurements to assess the impacts of developmental queen exposure on colony performance. There were no statistically significant differences when comparing total brood area and wax production between colonies headed by queens reared in difference pesticide exposure environments. Colonies headed by experimental queens reared in colonies fed treated pollen supplement had lower brood viability relative to those reared in control colonies (Mixed model Fixed effects test, n = 250, Pollen treatment: F Ratio_(1,20.11)_ = 7.12, *P* < 0.05) (Fig. 5). At the conclusion of the study, varroa mite counts in colonies established by queens reared in different wax and pollen exposure environments did not vary between treatment (Mixed model Fixed effects test, n = 27; Wax: F_(1,22.4)_ = 4.15, *P* = 0.053; Pollen: F_(1,4.2)_ = 1.22, *P* = 0.33).

## Discussion

The treated pollen supplement administered to colonies prior to and during queen rearing was found to contain an HQ of 1653 (Supplemental Table 2) and represents a substantial level of pesticide hazard when compared to residue data from commercial colonies where only 15% of samples were found to have an HQ > 1000^29^. Queen development within colonies exposed to treated pollen resulted in lower acceptance of grafted larvae and reduced the survival of developing queens to emergence as adults (Fig. 2). Curiously, despite the observed phenotypic effects resulting from our treated pollen supplement on queen survival during development, we did not observe an increase in pesticide residues within the royal jelly (RJ) that was provisioned to the queen larvae (Supplemental Table 1 and 2). However, it has been observed that oral pesticide exposure can degrade the secretory glands of nurse bees that are used to synthesize royal jelly^31–34,36,52^. The lack of pesticides in RJ, concurrent with reductions in survival, indicates that our pesticide treatment likely had indirect effects on queen larvae (e.g., through reduced quality of RJ) rather than direct effects (i.e., exposure through contaminated RJ). Indeed, it has been reported that exposure to the same pesticide mixture used in this experiment heavily influenced the nutritional composition of RJ produced by exposed colonies without an increase of pesticide residues^37^. Consequently, the phenotypic effects reported in the present study are likely the result of pesticides having an impact on RJ synthesis, nutrition, or both and not a result of direct pesticide exposure. Workers terminate brood rearing in nutritionally stressed circumstances^53^, and additional selection against unfit queens may have also resulted in reduced queen production in colonies exposed to pesticide treated pollen. It is adaptive for workers to divest resources from developmentally stressed queens, and it has been observed that caged queen cells (removed from worker-mediated selection) yield smaller queens relative to cells that can be accessed by workers^54^. We observed no differences when comparing viral loads between workers from colonies fed treated or untreated pollen (Supplemental Table 3) during our experiment, no significant differences were detected when comparing queen cell production or survival between wax treatment groups.

**Figure 4:**
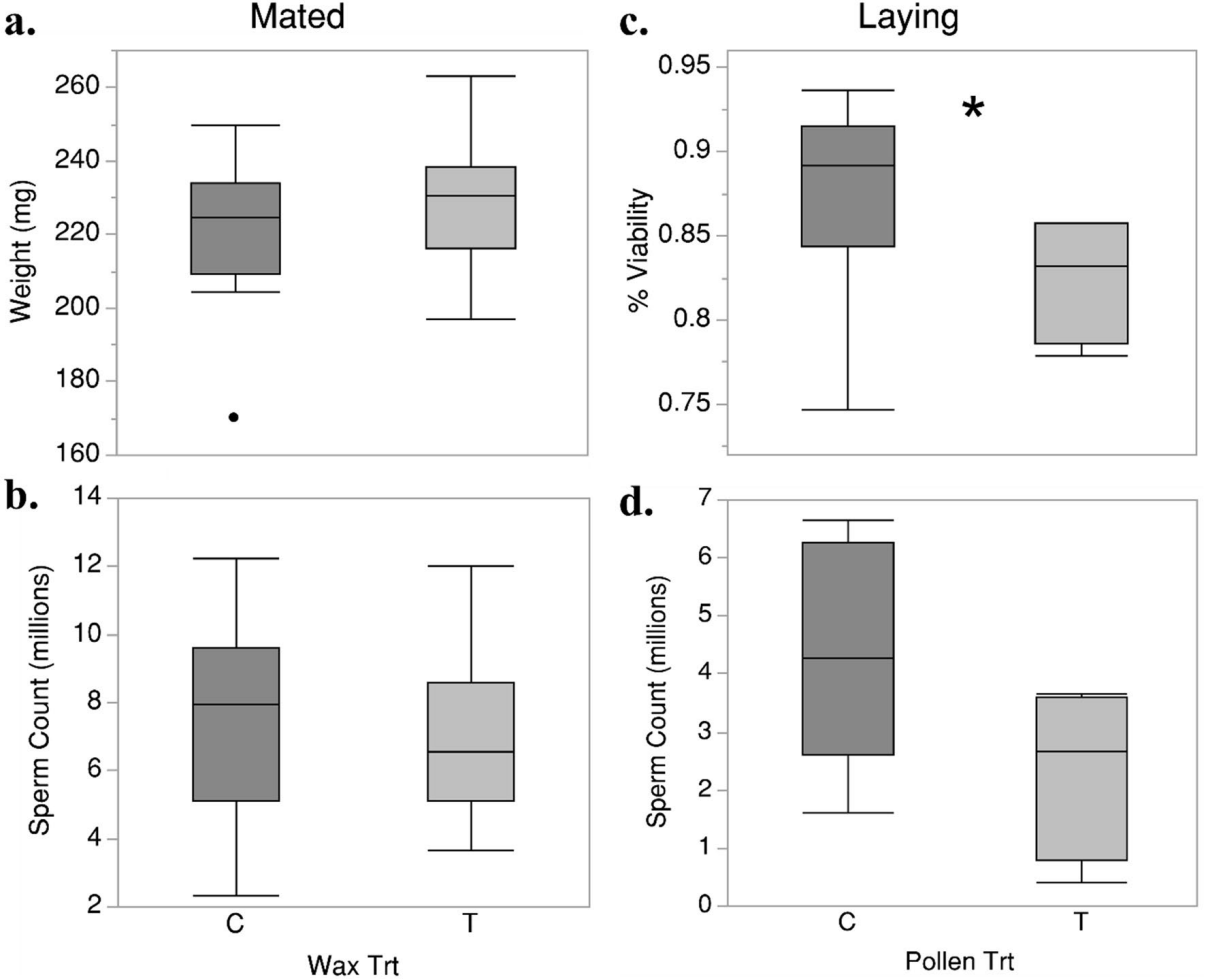
A comparison between queens reared treated (T) and control (C) wax cups and colonies, *P < 0.05. Mated queens are those which were sampled following the initiation of egg laying (**a**,**b**) (Control, n = 18. Treated n = 17). Laying queens were those sampled at the end of the colony growth period (**c**,**d**) (Control, n = 14; Treated n = 4).

**Figure 5:**
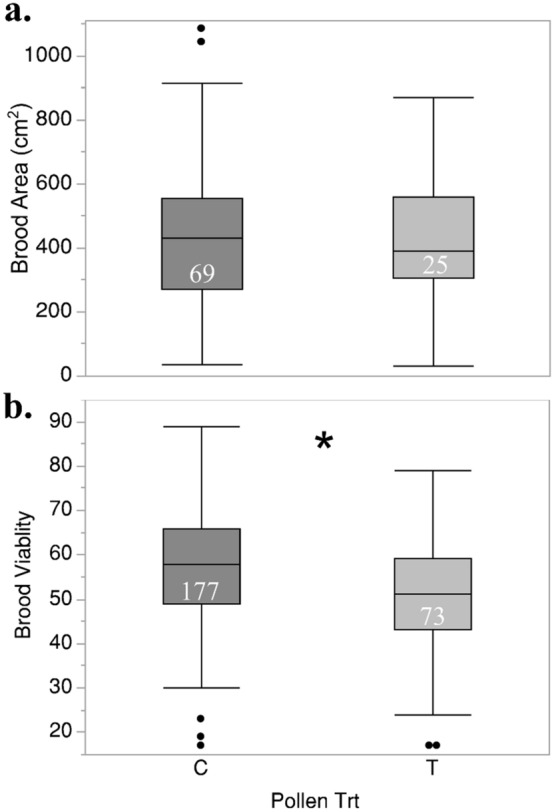
A comparison of cumulative brood area (**a**) and brood viability (**b**) between colonies headed by queens reared in colonies administered control (C) or treated (T) pollen supplements, *P < 0.05. The number of observations for each is shown inside each plot.

In total, 160 adult queens were introduced into mini “nucleus” colonies for mating from two grafting rounds. No significant differences in mating success, as determined by the successful initiation of egg laying, were found when comparing queens across wax and pollen treatments. A subsample of queens reared in control pollen colonies (n = 35) were analyzed immediately post-mating, and we found that queens reared in our treated wax had similar morphology and stored-sperm quality relative to those reared in wax impregnated only with solvent (Fig. 4a). Because of low sample size (as a result of the poor rearing success), no comparisons were made investigating the effect of the pollen treatment at this stage, electing to introduce all 39 experimental queens into artificial swarms to measure the impacts of developmental exposure on newly established colony growth. We observed no effects of queen rearing pesticide exposure environment on the recorded monthly measures of brood area and total wax construction, although we did observe a statistically significant reduction in brood viability between those queens reared in colonies fed treated pollen relative to controls (Fig. 5). Pupal brood pattern has been often associated with queen quality and can be attributed to inbreeding or low diversity^17^. Additionally, it has been previously observed that topical pesticide exposure directly on adult queens^20^ and colony-level exposure to pesticides spiked sugar syrup^39^ can also reduce brood viability. The differences in brood viability between pollen treatment groups observed in this study demonstrate a downstream impact of queen developmental conditions on later established colonies. However, colony brood viability is not only a result of queen health but also a variety of colony-level factors including disease infection^19^, pesticide residues in wax comb^55^, and nutritional status^53^. Moreover, the reliability of brood pattern as a proxy for queen health has been called into question^56^, and more work is needed to better understand the relationship between queen development and colony conditions on brood pattern. Curiously, we observed that colonies headed by queens reared in treated wax had a lower number of varroa mites per bee and this effect was marginally significant (*P* = 0.053). However, the mechanism by which a queens developmental pesticide exposure environment could influence varroa infestation is unknown and this finding is likely spurious.

At the conclusion of the colony growth period (after mated queens were fully established in colonies), queens were again sampled for reproductive health (n = 18). We found that queens produced by colonies exposed to contaminated pollen had significant reduced sperm viability and lower sperm counts (Fig. 3b). The ability of queens to maintain large numbers of healthy sperm in their spermatheca is an important facet of queen quality^15,46^, and reductions in sperm viability following exposure to pesticides have been previously reported^21,26,38^ but the specific mechanism driving this relationship is unclear. Antioxidants^57^ and ATP-independent heat shock proteins have been previously found to be important for sperm longevity, and reactive oxygen species produced following development in pesticide exposed colonies could present a challenge to the sperm which later occupy queen’s spermatheca^58^. We also found that developmental exposure might manifest in the quantity of sperm stored within a queen’s spermatheca. When stored sperm counts were compared at the end of the colony growth period, we observed a significant effect of pollen treatment (one-tailed t_16_ = − 1.89, *P* < 0.05). Lower numbers of stored sperm have also been previously reported in other works measuring the impacts of pesticide on queens^21,26^, and our current finding likely arises from differences in queen physiology rather than from differences in mating number or drone sperm quality. Upon reaching reproductive maturity, a single drone contains ~ 7 million spermatozoa^59^ and we observed an average mating number of 21 drones (*n*_o_) for queens raised in treated pollen colonies; these same queens were also found to have an average sperm count of 2.3 million spermatozoa. As a result, it is likely that differences in sperm count result from differences in sperm migration into the spermatheca following mating events^60^ or, perhaps, as a consequence of more rapid sperm depletion during egg fertilization and laying. The underlying mechanism driving differences in sperm count and viability in response to development within pesticide exposed colonies should be examined. No differences were found when comparing disease among laying queens at the end of the experiment (Supplemental Table 4).

Queens mate with multiple males, and the number of mates by a given queen can have a dramatic influence on colony phenotype; a genetically diverse worker population can help improve a colonies’ disease response and reduce inbreeding^12,14^. Despite trends towards lower mating numbers in queens reared in treated pollen colonies, we observed no significant differences when looking at the effective mating number (*m*_e_) or observed mating number (*N*_o_) of queens between treatment groups (Fig. 3) and as a result we cannot fully attribute this trend to our pesticide treatment. A previous study comparing mating numbers between queens reared in colonies exposed to neonicotinoids found significant reductions in the *N*_o_ and *m*_e_ for treated queens^26^. Alternatively, a study investigating the impacts of a contact exposure to high doses of a two miticides observed an increase in mating number in response to the treatment^23^. This discrepancy could arise from the different compounds tested and from the aforementioned indirect effects of colony-level oral exposure on developmental queen nutrition as opposed to direct pesticide exposure. Future works should report the mechanism by which these differing exposures result in diverging responses on mating number.

We found no statistically significant impacts of our wax treatment on queen survival, mating health, or downstream colony growth. Additionally, we saw no differences when comparing colony growth between queens reared in treated and untreated wax. Contrary to RJ from colonies administered pesticide-treated pollen, residue analysis of RJ samples from cells formed from treated beeswax did show some degree of residue transfer at a concentration of about 10% for some compounds relative to residues in wax (Supplemental Table 1). Based on our residue analysis, we calculated our experimental treated wax to have an HQ of 2606 (Supplemental Table 2). This value represents a near-average exposure compared to previously reported commercial colony wax contamination levels (mean HQ = 2255)^29^ and could possibly explain the minimal effects that our treated wax had on developing queens. Higher levels of pesticide contamination in wax have been found to impact queen health, and previous works focusing on miticides have demonstrated the potential impacts contaminated wax can have on queen development. One series of experiments measuring the impacts of coumaphos impregnated wax during queen rearing found that the 100 ppm dose level (HQ = 16,863) significantly lowered grafting acceptance and queen pupal weight^22^ but no differences were detected when examining sperm concentration^61^. Alternatively, a study measuring the combined effects of developmental exposure to 94 ppm coumaphos and 204 ppm tau-fluvalinate in wax (HQ_Total_ = 63,074) found significant reductions in sperm count and viability in mated queens^23^. These works demonstrate that despite no direct effects resulting from our wax exposure on queen health, higher levels of wax contamination than typically found in field conditions can indirectly lead to reductions in queen survival and reproduction.

Our work demonstrates how highly contaminated pollen can negatively affect queen reproductive quality. Queen producers adjacent to agriculturally intensive areas with elevated pesticide use on pollinator attractive crops may suffer the effects of exposure when it comes to producing large numbers of viable queens. Moreover, this highlights how colony level pesticide exposure may pose a challenge during the sensitive period following the loss of a queen. It has been previously reported that colonies containing highly contaminated wax (HQ = 6500) had an increased incident of queen loss^29^. These findings also highlight how developmental exposure to pesticide contaminated pollen can have long-lasting effects on queens and how developmental stress can later influence established colonies. Queens surviving developmental bottlenecks following colony-level exposure were still compromised and, despite mating successfully, headed colonies with reduced brood viability. Although, our sample size was greatly reduced through attrition by the end of the experiment, our work supports the notion that individual health outcomes resulting from developmental stress on queens have the potential to inflict a collective cost on colony health. As such, future works should continue to characterize how stressors shape developmental conditions in honey bees.

## Supplementary information

Supplementary Information.

## References

[1] Polderman TJC (2015). Meta-analysis of the heritability of human traits based on fifty years of twin studies. Nat. Genet..

[2] Wild CP (2005). Complementing the genome with an ‘exposome’: The outstanding challenge of environmental exposure measurement in molecular epidemiology. Cancer Epidemiol. Biomark. Prev..

[3] Schug TT, Janesick A, Blumberg B, Heindel JJ (2012). Endocrine disrupting chemicals and disease susceptibility. J. Steroid. Biochem. Mol. Biol..

[4] Gluckman PD, Hanson MA, Cooper C, Thornburg KL (2008). Effect of in utero and early-life conditions on adult health and disease. N. Engl. J. Med..

[5] Wilson-Sanders SE (2011). Invertebrate models for biomedical research, testing, and education. ILAR J..

[6] Klein A (2007). Importance of pollinators in changing landscapes for world crops. Proc. R. Soc. B.

[7] Johnson RM (2014). Honey bee toxicology. Annu. Rev. Entomol..

[8] O’Neal ST, Anderson TD, Wu-Smart JY (2018). Interactions between pesticides and pathogen susceptibility in honey bees. Curr. Opin. Insect Sci..

[9] Seeley TD (1989). The honey bee colony as a superorganism. Am. Sci..

[10] VanEngelsdorp D, Meixner MD (2010). A historical review of managed honey bee populations in Europe and the United States and the factors that may affect them. J. Invertebr. Pathol..

[11] Winston ML (1987). The biology of the honey bee.

[12] Seeley TD, Tarpy DR (2007). Queen promiscuity lowers disease within honeybee colonies. Proc. Biol. Sci..

[13] Tarpy DR, Seeley TD (2006). Lower disease infections in honeybee (*Apis mellifera*) colonies headed by polyandrous vs monandrous queens. Naturwissenschaften.

[14] Tarpy DR (2003). Genetic diversity within honeybee colonies prevents severe infections and promotes colony growth. Proc. R. Soc. Lond. B.

[15] Baer B, Collins J, Maalaps K, den Boer SPA (2016). Sperm use economy of honeybee (*Apis mellifera*) queens. Ecol. Evol..

[16] Tarpy DR, Vanengelsdorp D, Pettis JS (2013). Genetic diversity affects colony survivorship in commercial honey bee colonies. Naturwissenschaften.

[17] Tarpy DR, Keller JJ, Caren JR, Delaney DA (2012). Assessing the mating ‘health’ of commercial honey bee queens. J. Econ. Entomol..

[18] Kulhanek K (2017). A national survey of managed honey bee 2015–2016 annual colony losses in the USA. J. Apic. Res..

[19] VanEngelsdorp D, Tarpy DR, Lengerich EJ, Pettis JS (2013). Idiopathic brood disease syndrome and queen events as precursors of colony mortality in migratory beekeeping operations in the eastern United States. Prev. Vet. Med..

[20] Akinwande KL, Lizette D, Johnson RM, Siegfried BD, Ellis MD (2014). Effect of amitraz on queen hone bee egg and brood development. Mellifera.

[21] Chaimanee V, Evans JD, Chen Y, Jackson C, Pettis JS (2016). Sperm viability and gene expression in honey bee queens (*Apis mellifera*) following exposure to the neonicotinoid insecticide imidacloprid and the organophosphate acaricide coumaphos. J. Insect Physiol..

[22] Pettis J, Collins A, Wilbanks R, Feldlaufer MF (2004). Effects of coumaphos on queen rearing in the honey bee, *Apis mellifera*. Apidologie.

[23] Rangel J, Tarpy DR (2015). The combined effects of miticides on the mating health of honey bee (*Apis mellifera* L.) queens. J. Apic. Res..

[24] Brandt A, Grikscheit K, Siede R, Grosse R, Doris M, Büchler R (2017). Immunosuppression in honeybee queens by the neonicotinoids thiacloprid and clothianidin. Sci. Rep..

[25] Forfert N (2017). Neonicotinoid pesticides can reduce honeybee colony genetic diversity. PLoS ONE.

[26] Williams GR (2015). Neonicotinoid pesticides severely affect honey bee queens. Sci. Rep..

[27] Bonzini S, Tremolada P, Bernardinelli I, Colombo M, Vighi M (2011). Predicting pesticide fate in the hive (part 1): Experimentally determined τ-fluvalinate residues in bees, honey and wax. Apidologie.

[28] Tremolada P, Bernardinelli I, Colombo M, Spreafico M, Vighi M (2004). Coumaphos distribution in the hive ecosystem: Case study for modeling applications. Ecotoxicology.

[29] Traynor KS, Pettis JS, Tarpy DR, Mullin CA, Frazier JL, Frazier M, VanEngelsdorp D (2016). In-hive pesticide exposome: Assessing risks to migratory honey bees from in-hive pesticide contamination in the Eastern United States. Sci. Rep..

[30] Zaluski R, Justulin LA, Orsi RO (2017). Field-relevant doses of the systemic insecticide fipronil and fungicide pyraclostrobin impair mandibular and hypopharyngeal glands in nurse honeybees (*Apis mellifera*). Sci. Rep..

[31] Renzi MT (2016). Combined effect of pollen quality and thiamethoxam on hypopharyngeal gland development and protein content in *Apis mellifera*. Apidologie.

[32] Hatjina F (2013). Sublethal doses of imidacloprid decreased size of hypopharyngeal glands and respiratory rhythm of honeybees in vivo. Apidologie.

[33] Faita MR, Oliveira EM, Alves VV, Orth AI, Nodari RO (2018). Changes in hypopharyngeal glands of nurse bees (*Apis mellifera*) induced by pollen-containing sublethal doses of the herbicide roundup. Chemosphere.

[34] Böhme F, Bischoff G, Zebitz CP, Rosenkranz P, Wallner K (2016). Chronic exposure of honeybees, *Apis mellifera* (Hymenoptera: Apidae), to a pesticide mixture in realistic field exposure rates. Apidologie.

[35] Alaux C (2010). Interactions between Nosema microspores and a neonicotinoid weaken honeybees (*Apis mellifera*). Environ. Microbiol..

[36] Berenbaum MR (2019). Honey bees and environmental stress : Toxicologic pathology of a superorganism. Toxicol. Pathol..

[37] Milone JP, Chakrabarti P, Sagili RR, Tarpy DR (2021). Colony-level pesticide exposure affects honey bee (*Apis mellifera* L.) royal jelly production and nutritional composition. Chemosphere.

[38] Rangel J, Keller JJ, Tarpy DR (2013). The effects of honey bee (*Apis mellifera* L.) queen reproductive potential on colony growth. Insectes Soc..

[39] Wu-smart J, Spivak M (2016). Sub-lethal effects of dietary neonicotinoid insecticide exposure on honey bee queen fecundity and colony development. Nat. Publ. Gr..

[40] Stoner KA, Eitzer BD (2013). Using a hazard quotient to evaluate pesticide residues detected in pollen trapped from honey bees (*Apis mellifera*) in Connecticut. PLoS ONE.

[41] Fries I, Wallner K, Rosenkranz P (1998). Effects on *Varroa jacobsoni* from acaricides in beeswax. J. Apic. Res..

[42] Laidlaw HH, Page RE (1997). Queen Rearing and Bee Breeding.

[43] Collins AM, Donoghue AM (1999). Viability assessment of honey bee, *Apis mellifera*, sperm using dual flurescent staining. Theriogenology.

[44] Withrow JM, Tarpy DR (2018). Cryptic, “royal” subfamilies in honey bee (*Apis mellifera*) colonies. PLoS ONE.

[45] Evans JD (2015). Standard methods for molecular research in *Apis mellifera*. J. Apic. Res..

[46] Delaney DA, Keller JJ, Caren JR, Tarpy DR (2011). The physical, insemination, and reproductive quality of honey bee queens (*Apis mellifera* L.). Apidologie.

[47] Wang J (2004). Sibship reconstruction from genetic data with typing errors. Genetics.

[48] Tarpy DR, Nielsen R, Nielsen DI (2004). A scientific note on the revised estimates of effective paternity frequency in Apis. Insectes Soc..

[49] Delaplane KS, Van Der Steen J, Guzman-novoa E (2013). Standard methods for estimating strength parameters of *Apis mellifera* colonies. J. Apic. Res..

[50] Jong DE, AndreaRoma DE, Gonçalves LS (1982). A comparative analysis of shaking solutions for the detection of *Varroa jacobsoni* on adult honeybees. Apidologie.

[51] Mullin CA (2010). High levels of miticides and agrochemicals in North American apiaries: Implications for honey bee health. PLoS ONE.

[52] Zaluski R, Justulin LA, de Oliveira Orsi R (2017). Field-relevant doses of the systemic insecticide fipronil and fungicide pyraclostrobin impair mandibular and hypopharyngeal glands in nurse honeybees (*Apis mellifera*). Sci. Rep..

[53] Brodschneider R, Crailsheim K (2010). Nutrition and health in honey bees. Apidologie.

[54] Tarpy DR, Linksvayer TA (2016). Honey bee colonies regulate queen reproductive traits by controlling which queens survive to adulthood. Insectes Soc..

[55] Wu JY, Smart MD, Anelli CM, Sheppard WS (2012). Honey bees (*Apis mellifera*) reared in brood combs containing high levels of pesticide residues exhibit increased susceptibility to Nosema (Microsporidia) infection. J. Invertebr. Pathol..

[56] Lee KV, Goblirsch M, Mcdermott E, Tarpy DR, Spivak M (2019). Is the brood pattern within a honey bee colony a reliable indicator of queen quality?. Insects.

[57] Collins AM, Williams V, Evans JD (2004). Sperm storage and antioxidative enzyme expression in the honey bee, *Apis mellifera*. Insect Mol. Biol..

[58] McAfee A, Chapman A, Higo H, Underwood R, Milone J, Foster LJ, Guarna MM, Tarpy DR, Pettis JS (2020). Vulnerability of honey bee queens to heat-induced loss of fertility. Nat. Sustain..

[59] Metz BN, Tarpy DR (2019). Reproductive senescence in drones of the honey bee (*Apis mellifera*). Insects.

[60] Page R (1986). Sperm utilization in social insects. Annu. Rev. Entomol..

[61] Collins AM, Pettis JS, Wilbanks R, Feldlaufer MF (2004). Performance of honey bee (*Apis mellifera*) queens reared in beeswax cells impregnated with coumaphos. J. Apic. Res..

