# Supplementary material for: Effects of developmental exposure to pesticides in wax and pollen on honey bee (Apis mellifera) queen reproductive phenotypes
Source: Sci Rep. 2021 Jan 13;11:1020. doi: 10.1038/s41598-020-80446-3 (PMC7806648; doi:10.1038/s41598-020-80446-3)
Supplement: Supplementary file 1 — Supplementary Information. [file 41598_2020_80446_MOESM1_ESM.docx]

Effects of developmental exposure to pesticides in wax and pollen on honey bee (*Apis mellifera*) queen reproductive phenotypes

Joseph P. Milone and David R. Tarpy

**Supplemental Information**

| Pesticide | LD50 (µg/bee) | Treated wax | | Control wax | | |
| --- | --- | --- | --- | --- | --- | --- |
|  |  | T Pollen | C pollen | | T Pollen | C pollen |
| Atrazine | 98.5 | ND | 0.04 | | ND | ND |
| Azoxystrobin | 112.00 | 0.02 | 0.02 | | ND | ND |
| Chlorothalonil | 111 | 12.70 | 15.68 | | ND | ND |
| Chlorpyrifos | 0.0762 | 118.11 | 170.60 | | ND | ND |
| Coumaphos | 5.93 | 28.67 | 26.98 | | 1.35 | 1.01 |
| Coumaphos oxon | 5.93 | 0.24 | 0.08 | | 0.12 | ND |
| DEET | - | (7ppb) | (8ppb) | | (8ppb) | (8ppb) |
| 2,4-DMPF | 75.00 | 51.47 | 50.00 | | 0.24 | 0.17 |
| Fluvalinate | 4.32 | 53.47 | 43.75 | | ND | ND |
| Metolachlor | 126 | ND | ND | | 0.20 | ND |
| Thymol | - | (989ppb) | (955ppb) | | (2ppb) | (1,290ppb) |
|  | **Total HQ:** | **264.68** | **307.16** | | **1.91** | **1.19** |

**Supplemental Table 1.** Hazard Quotient values calculated from residue detections in royal jelly from colonies exposed to treated ( T ) or control ( C ) pollen supplement and harvested from wax queen cups made from treated and untreated beeswax. LD_50_ values for each compound were used from Traynor et al. 2016 **^1^**. Any trace detections had their HQ value calculated using the compound’s limit of detection in order to determine the worst case HQ. No honey bee LD_50_ has been reported for DEET and thymol is non-toxic to honey bees. As a result, we report the detected residue concentrations for these compounds. *2,4-*DMPF and coumaphos oxon are metabolites of the pesticides amitraz and coumaphos respectively.

| Pesticide | LD50 (µg/bee) | Pollen detections (ppb) | | | | Wax detections (ppb) | | | |
| --- | --- | --- | --- | --- | --- | --- | --- | --- | --- |
|  |  | Treated pollen | HQ | Control pollen | HQ | Treated wax | HQ | Control wax | HQ |
|  |  |  |  |  |  |  |  |  |  |
|  |  |  |  |  |  |  |  |  |  |
| Atrazine | 98.50 | 25.0 | 0.25 | ND | 0 | 61.0 | 0.62 | ND |  |
| Azoxystrobin | 112.00 | 78.0 | 0.70 | ND | 0 | 56.0 | 0.50 | 12.0 | 0.1 |
| Carbaryl | 0.442 | 368.0 | 832.58 | ND | 0 | ND | 0.00 | ND | 0 |
| Carbendazim | 756 | TRACE | 0.01 | TRACE | 0.01 | ND | 0.00 | ND | 0 |
| Chlorothalonil | 111.00 | 16,000.0 | 144.14 | ND | 0 | 13,000.0 | 117.12 | ND | 0 |
| Chlorpyrifos | 0.08 | 20.0 | 262.47 | ND | 0 | 113.0 | 1,482.94 | ND | 0 |
| Coumaphos | 5.93 | 1,870.0 | 315.35 | ND | 0 | 1,680.0 | 283.31 | 25.0 | 4.2 |
| Coumaphos oxon | 5.93 | ND | 0 | ND | 0 | 12.0 | 2.02 | 10.0 | 1.7 |
| DEET | - | ND | 0 | ND | 0 | 92.0 | N/A | 98.0 | N/A |
| 2,4-DMPF | 75.00 | 9.0 | 0.12 | 14.0 | 0.19 | 32,900.0 | 438.67 | 4.0 | 0.1 |
| Fenpropathrin | 0.05 | 0.0 | 0 | ND | 0 | ND | 0.00 | ND | 0 |
| Fluoxastrobin | 843.00 | ND | 0 | ND | 0 | 11.0 | 0.01 | 11.0 | 0 |
| Flutriafol | 2.00 | ND | 0 | ND | 0 | 29.0 | 14.50 | 27.0 | 13.5 |
| Fluvalinate | 4.32 | 415.0 | 96.06 | ND | 0 | 1,100.0 | 254.63 | 53.0 | 12.3 |
| Pendimethalin | 74.90 | 105.0 | 1.40 | ND | 0 | TRACE | 1.87 | ND | 0 |
| Thymol | - | 89.0 | N/A | ND | 0 | 2,200.0 | N/A | 2,500.0 | N/A |
|  |  | **HQ Total:** | **1653.1** |  | **0.2** |  | **2596.2** |  | **31.9** |

**Supplemental Table 2.** Residues detected in treated and control wax used for queen cells and pollen supplement administered to colonies. Hazard Quotients (HQ) were calculated according to Stoner et al. 2012**^2^** and were summed to give a total HQ for each matrix. LD_50_ values for each compound were used from Traynor et al. 2016**^1^**. Any trace detections had their HQ value calculated using the compound’s limit of detection in order to determine the worst case HQ. No honey bee LD_50_ has been reported for DEET and thymol is non-toxic to honey bees. *2,4-*DMPF and coumaphos oxon are metabolites of the pesticides amitraz and coumaphos, respectively.

| Colony | Trt | CBPV | | DWVA | | IAPV | | LSV | | Nosema | | Trypanosomes | |
| --- | --- | --- | --- | --- | --- | --- | --- | --- | --- | --- | --- | --- | --- |
|  |  | Pre | Post | Pre | Post | Pre | Post | Pre | Post | Pre | Post | Pre | Post |
| 1 | C | 0 | 21,131 | 0 | 25,324 | 0 | 0 | 508,778 | 351,021 | 82,680 | 18,130 | 8,014 | 2,105 |
| 2 | C | 0 | 0 | 0 | 0 | 0 | 0 | 18,223 | 93,163 | 230,292 | 21,621 | 3,025 | 867 |
| 3 | T | 0 | 0 | 0 | 0 | 0 | 1372 | 39,146 | 302,865 | 256,06 | 0 | 0 | 0 |
| 4 | C | 0 | 0 | 0 | 0 | 0 | 304 | 55,972 | 308,497 | 5,825 | 768 | 0 | 1,773 |
| 5 | T | 0 | 0 | 0 | 0 | 0 | 0 | 484,330 | 116,843 | 21,575 | 0 | 0 | 19,571 |
| 6 | T | 0 | 0 | 0 | 241 | 0 | 0 | 4,392 | 281,137 | 11,528 | 1,800 | 0 | 6,490 |

**Supplemental Table 3.** Pathogen levels presented in genome equivalents for worker bees collected prior to (Pre) and at the conclusion (Post) of feeding with a treated ( T ) or control ( C ) pollen supplement. CBPV- Chronic bee paralysis virus, DWVA- deformed wing virus A, IAPV- Israeli acute paralysis virus, and LSV- Lake Sinai virus. Universal primers were used to detect Nosema and Trypanosomes. The following pathogens were screened for but not detected: Acute be paralysis virus, deformed wing virus B, and Black queen cell virus.

| Queen | Pollen treatment | Wax Treatment | DWVA | Nosema |
| --- | --- | --- | --- | --- |
| 1 | C | T | 0 | 0 |
| 2 | C | T | 323,105 | 0 |
| 3 | C | C | 0 | 0 |
| 4 | C | C | 331,534 | 0 |
| 5 | C | T | 0 | 0 |
| 6 | C | T | 98,809.6 | 0 |
| 7 | C | C | 0 | 0 |
| 8 | C | C | 170,835 | 0 |
| 9 | C | C | 0 | 0 |
| 10 | C | C | 0 | 0 |
| 11 | C | T | 153.685 | 0 |
| 12 | C | T | 0 | 0 |
| 13 | C | T | 0 | 0 |
| 14 | C | C | 140,634 | 0 |
| 15 | T | C | 1,376.16 | 89.3982 |
| 16 | T | T | 829.866 | 0 |
| 17 | T | T | 0 | 0 |
| 18 | T | T | 163,962 | 0 |

**Supplemental Table 4.** Pathogen levels presented in genome equivalents from queens at the conclusion of the experiment. T and C are used to indicate treatment groups for developmental rearing environment. DWVA- deformed wing virus A. The following pathogens were screened for but not detected: Israeli acute paralysis virus, Lake Sinai virus, Trypanosomes, Acute bee paralysis virus, Deformed wing virus B, Black queen cell virus, and Chronic bee paralysis virus. Universal primers were used to detect Nosema and Trypanosomes.

| Target | Type | Forward sequence | Reverse sequence |
| --- | --- | --- | --- |
| Black Queen Cell Virus | Virus | CGAAGCGTTTTCCGTGG | GCTGTCGAGAGTCAGAGTT |
| Chronic Bee Paralysis Virus | Virus | ACTGCTGCCCTCGATAG | TGTGTTGAGGCAGGTTGG |
| Acute Bee Paralysis Virus | Virus | TCCCAAGATTGGAATAAGACAGTTAG | TTCCATAATGCAAACATTCAAAGATCC |
| Israel Acute Paralysis Virus | Virus | GCTAATACCAAGACACCAATCACGGACC | TCTCGACCCTGAGCATCTGTG |
| Lake Sinai Virus | Virus | TCATCCCAAGAGAACCACT | CGCGTGTGCATGGAA |
| Nosema universal | Protozoan | AGCAGCCGCGGTAATACTTGTTC | GTTCGTCCAGTCAGGGTCGT |
| Trypanosome universal | Protozoan | GAGTGTGGCAGGACTACCC | TGCACCAACCACGAAATGA |
| Deformed Wing Virus A | Virus | GTCTTGTGGATGAAGGTTATATAACTGG | TCCGTAGAAAGCCGAGTTG |
| Deformed Wing Virus B | Virus | ACCAACGCGTGTCGTTCCTG | ACAAGTGGTTGGTCCCGTCG |
| Actin 1 | Reference | TTGGCTGGCCGTGATTTGAC | GGACAACGGAATCTTTCGTTACCAAT |
| APO 28S | Reference | TGGTTCCCTCCGAAGTTTCCCTCAG | GCAAGCCAGAGATCTCACCCATTTA |

**Supplemental Table 5.** Forward and reverse primer sequences used for quantifying disease in workers from cell builder colonies and queens post laying.

**References**

1. Traynor, K. S., Pettis, J. S., Tarpy, D. R., Mullin, C. A., Frazier, J. L., Frazier, M. & VanEngelsdorp, D. In-hive Pesticide Exposome: Assessing risks to migratory honey bees from in-hive pesticide contamination in the Eastern United States. Sci. Rep. 6, 33207 (2016).
2. Stoner, K. A. & Eitzer, B. D. Using a Hazard Quotient to Evaluate Pesticide Residues Detected in Pollen Trapped from Honey Bees (Apis mellifera) in Connecticut. *PLoS One* 8, e77550 (2013).
